# Numerical and Experimental Investigation of the Hemodynamic Performance of Bifurcated Stent Grafts with Various Torsion Angles

**DOI:** 10.1038/s41598-018-31015-2

**Published:** 2018-08-22

**Authors:** Ming Liu, Anqiang Sun, Xiaoyan Deng

**Affiliations:** 1 Key Laboratory for Biomechanics and Mechanobiology of Ministry of Education, School of Biological Science and Medical Engineering, Beihang University, Beijing, 100083 China; 2 Beijing Advanced Innovation Centre for Biomedical Engineering, Beihang University, Beijing, 102402 China

## Abstract

The “crossed limbs” strategy for bifurcated stent grafts (BSGs) is widely employed when abdominal aortic aneurysm (AAA) patients have unfavorable neck or highly splayed iliac arteries. Helical flow is regarded as a typical flow pattern within the human arterial system and is believed to have the positive physiological effects of inhibiting thrombosis formation and atherosclerosis. The “crossed limbs” strategy may induce helical flow and improve the stent graft outcome. To verify the performance of this strategy by considering hemodynamics, we constructed a series of idealized BSGs with various torsion angles and evaluated the hemodynamic performance, including the helical strength, time-averaged wall shear stress (TAWSS), oscillatory shear index, relative resident time (RRT), and displacement force. Our numerical results indicate that an increased torsion angle enhances the helicity strength at the iliac outlets. However, with increasing torsion angle, the TAWSS in the iliac graft decreases and the RRT increases. In addition, our numerical simulations and *in vitro* experiments reveal that the displacement force increases gradually with increasing torsion angle. In summary, the “crossed limbs” strategy may have benefits for AAA treatment in terms of helical flow, but because of the unfavorable hemodynamic performance verified by analyzing the hemodynamic indicators, the risk of stent graft migration increases with increasing torsion angle. Therefore, the “crossed limbs” strategy should be carefully employed in surgical AAA treatment.

## Introduction

During endovascular aneurysm repair (EVAR), the insertion of a bifurcated stent graft (BSG) into an abdominal aortic aneurysm (AAA) is more difficult and time-consuming when the patient has severe aneurysm neck angulation or highly splayed iliac arteries^1,2^. To address this problem, BSGs with intentionally “crossed limbs” are regularly employed^3–5^. With this deployment strategy, the cannulation time due to adverse anatomy, as well as the occurrence rate of complications, can be substantially reduced^4–6^. Stent graft thrombosis and migration are regarded as two typical complications of EVAR^1,7^. Stent graft thrombosis can occur as a result of stent graft thrombo-emboli and can lead to graft limb occlusion and lower-extremity ischemia. Migration is believed to be a long-developing process, which leads to endoleak, pressurization within the aneurysm sac, and AAA rupture.

Studies have revealed that helical flow is widely observed in the human arterial system^8,9^. Several researchers have investigated the beneficial effects of helical blood flow. Morbiducci *et al*. suggested that helical flow could be an optimal flow form within the human arterial system for efficient perfusion^10^. Morbiducci *et al*. also quantitatively evaluated the helical phenomenon in aortic flow and demonstrated that it could inhibit excessive energy dissipation and ensure flow stability^11^. Gallo *et al*. suggested that helical flow suppresses flow separation within the carotid bifurcation, thus preventing atheroprone hemodynamics^12^. This helical flow has been demonstrated to have the important physiological functions of inhibiting atherosclerosis and thrombosis formation by affecting the transport of materials such as atherogenic lipids and oxygen^8,13^, thereby reducing platelet adhesion on the arterial wall^14,15^. Interestingly, several researchers have reported that the “crossed limbs” strategy may generate helical flow within the limb parts of BSGs^3,5^. Shek *et al*. investigated the effects of “crossed limbs” based on patient-specific models and found that helical flow could be induced by using this strategy^6^. We believe that if helical flow can indeed be created by using the “crossed limbs” strategy for BSGs, it will be highly advantageous for AAA repair due to the resulting reduction of the risk of graft limb occlusion induced by thrombosis formation within the stent graft. The migration behavior would also be influenced by this “crossed limbs” strategy.

Although the “crossed limbs” strategy has often been used in clinical practice, its performance with respect to hemodynamics has not been verified thus far. Because the long-term outcomes of stent graft repair for AAAs largely depend on hemodynamics performance^16–19^, we believe that it is necessary to study this issue further. Several investigations of the hemodynamic effects on the “crossed limbs” strategy have been conducted by performing computational simulations based on patient-specific models; however, the hemodynamic feature varies from individual to individual^3,6^. The common features of the “crossed limbs” strategy can be captured by using idealized models in which one geometric parameter is changed while the others are kept constant. BSG migration remains a difficult issue, and studies including computational simulations and *in vitro* experiments have been conducted to evaluate the displacement force and threshold forces causing stent graft migration^18,20^. However, *in vitro* experiments have remained necessary to verify the consequences of using the “crossed limbs” strategy in cases of migration risk. Therefore, in this study, we evaluated the hemodynamic performance of the “crossed limbs” strategy, including the flow pattern, helical strength, time-averaged wall shear stress (TAWSS), oscillatory shear index (OSI), and relative resident time (RRT). In addition, by conducting *in vitro* experiments, we analyzed the effects of the “crossed limbs” strategy on stent migration, which usually causes graft device failure, thus requiring re-intervention.

## Methods

### Geometry

**Figure 1:**
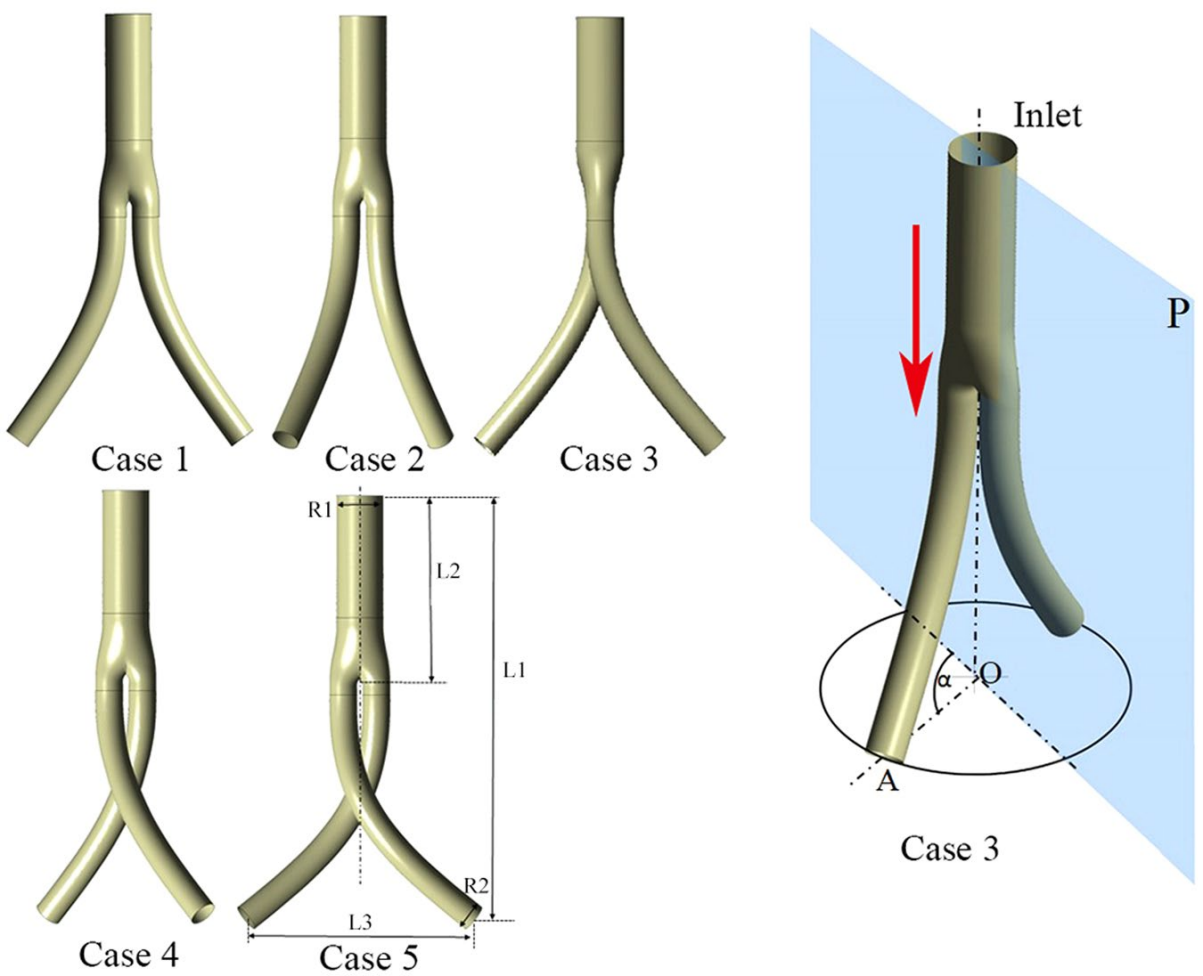
Five ideal BSGs with different torsion angles. The red arrow indicates the flow direction.

Five idealized BSG models with diverse torsion angles denoting the flow domain created by the “crossed limbs” EVAR technique were generated by SolidWorks (Solid Works Corp, Concord, MA) based on the BSG parameters adopted in the literature^18,19^. As depicted in Fig. 1, the torsion angle of the iliac artery graft is defined as the angle between the projection line OA on the basal surface and the symmetry plane P. OA is the line that connects the center point A of the left iliac graft outlet surface and the midpoint O of the bilateral iliac graft outlets, while P is the plane that traverses the initial bifurcation part of the bilateral iliac artery grafts. Specifically, the torsion angles of the five models were set to 0°, 45°, 90°, 135°, and 180°. The stent graft with a 0° torsion angle corresponds to a direct BSG, while that with a 180° torsion angle corresponds to a completely crossed BSG. All five models shared the same BSG trunk body. The total length L1 along the axis of the stent graft was 154.64 mm, including the stent graft trunk length L2 of 71 mm, and the side length L3 was 82 mm. The trunk diameter R1 was 17 mm, while the iliac graft diameter R2 was 10 mm.

### Governing equations

The simulations were performed based on the three-dimensional incompressible Navier-Stokes equation, together with the continuity equation:

1 $$\rho ((\partial \overrightarrow{\nu }/\partial t)+(\overrightarrow{\nu }\cdot \nabla )\overrightarrow{\nu })=-\,\nabla p+\nabla \tau $$

2 $${\rm{\nabla }}\cdot \overrightarrow{\nu }=0$$

where $\overrightarrow{\nu }$ is the fluid velocity vector, *p* is the pressure, *ρ* is the density of blood (1050 kg/m^3^), and ***τ*** is the stress tensor

3 $${\boldsymbol{\tau }}=2\eta (\dot{\gamma }){\bf{D}}$$

Here, **D** is the deformation tensor and $\dot{\gamma }$ is the shear rate. The blood viscosity *η* is a function of the shear rate, and the Carreau model can be used to incorporate the non-Newtonian characteristics of blood flow^21^:

4 $$\eta (\dot{\gamma })=({\eta }_{\infty })+({\eta }_{0}-{\eta }_{\infty }){[1+{(\lambda \dot{\gamma })}^{2}]}^{(({\rm{n}}-1)/2)}$$

where the infinite shear rate viscosity *η*_∞_ is 3.45 × 10^−3^ Pa · s, the zero shear rate *η*_0_ is 5.6 × 10^−2^ Pa · s, n = 0.3568, and the relaxation time constant *λ* is 3.313 s.

### Boundary conditions and meshing

**Figure 2:**
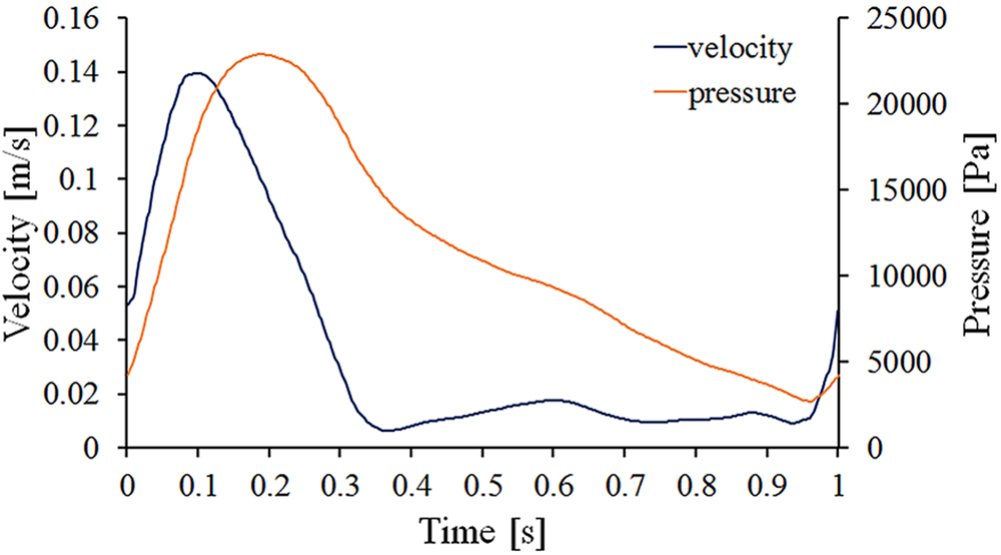
Imposed inlet velocity and outlet pressure waveforms.

In each case, a steady flow simulation was performed firstly; then, the steady flow solution was saved as the initial condition for the pulsatile simulations. An average velocity profile with an axial velocity of 0.04 m/s and a constant backpressure of 13,300 Pa was used at the inlet and outlets for the steady simulations. As shown in Fig. 2, for the pulsatile simulations, a time-dependent flat velocity waveform was imposed on the aorta inlet, while a pressure waveform was assigned at the bilateral outlets. As previously reported by Figuroa *et al*., the velocity flow data at the supraceliac level were measured by cine phase-contrast magnetic resonance imaging, and the pressure data were measured immediately after scanning^22^. The BSG wall was regarded as rigid and no-slip.

To ensure that the solutions of our simulations were mesh-independent, a total of four mesh sizes were obtained for validation (see Table 1). The conditions for mesh independence were defined as the differences of the area-weighted wall shear stress (WSS) of the iliac graft luminal surface and the velocity of the left outlet being less than 1% between two successive simulations under steady flow (see Table 1). For simplicity, the refined grids were directly used to simulate the pulsatile flow without any changes. As depicted in Table 1, when the number of cells in the mesh is greater than 1 × 10^6^, the differences in WSS and velocity can be ignored. The mesh size ranged from 0.05 mm to 0.6 mm, and the final mesh volume was about 1.7 × 10^6^ cells for each model.

**Table 1:** Mesh independence study results.

Mesh cells	Mesh nodes	Outlet velocity difference (%)	WSS difference (%)
964,256	267,528		
1,222,572	338,837	0.03%	0.05%
1,468,624	396,745	0.02%	0.05%
1,752,664	462,861	0.01%	0.02%

### Numerical schemes

The Navier-Stokes and continuity equations were solved by using ANSYS Fluent CFD (ANSYS Inc., Canonsburg, PA). During the calculation process, a total of 200 steps were performed for each cycle, and the time step was set to 0.005 s. A pressure-based solver was employed for the spatial discretization of the momentum. The threshold value for velocity and continuity residual detection was set to 1.0 × 10^−5^. To maintain the periodicity of the solutions, five pulsatile cycles were performed in total. The final solutions of the fifth cycle were extracted and compared in the analysis. MATLAB software was employed for the data analysis, and Tecplot software was used to display the hemodynamic indicators.

### Definitions of hemodynamic indicators

Helicity can be used to assess whether or not the velocity and vorticity vectors in a local region are aligned. Based on this information, the rotation direction of the helicity structures is indicated by the helicity sign^23^. In the present study, the helicity density *H*_*d*_ was employed to characterize the helical flow induced by the BSGs with different torsion angles and is defined as^12,24^

5 $${H}_{d}=\overrightarrow{\nu }\cdot (\nabla \times \overrightarrow{\nu })=\overrightarrow{\nu }\cdot \omega .$$

To evaluate the hemodynamic characteristics during the cardiac cycle quantitatively, hemodynamic indicators including the TAWSS, OSI, and RRT were defined and calculated based on the solutions of the pulsatile simulations. The TAWSS was used to measure the mean WSS throughout a cardiac cycle and was determined as follows:

6 $$TAWSS=\frac{1}{T}{\int }_{0}^{T}|{\bf{wss}}({\bf{s}},t)|\cdot dt.$$

In this formula, *T* is the cycle period, WSS is the instantaneous wall shear stress vector, and the position on the stent graft wall is represented by ***s***.

During one pulsatile cycle, the direction of the WSS varies instantaneously, and the definition of the OSI was used to elaborate this situation^25^:

7 $$OSI=\frac{1}{2}[1-(\frac{|{\int }_{0}^{T}{\boldsymbol{WSS}}({\bf{s}},t)\cdot dt|}{{\int }_{0}^{T}|{\boldsymbol{WSS}}({\bf{s}},t)|dt})].$$

The particles flowing in the blood may stagnate, leading to low and oscillatory shear stress on the graft wall. To assess the resident time of the flowing particles, the RRT index can be used^26^, which is quantified as

8 $$RRT=\frac{1}{\frac{1}{T}|{\int }_{0}^{T}{\boldsymbol{WSS}}({\bf{s}},t)\cdot dt|}.$$

The area-averaged values of the hemodynamic indicators were evaluated for quantitative comparison, using the following equation^27^:

9 $$\mu =\frac{{\sum }_{i=1}^{n}({A}_{i}\times {\varphi }_{i})}{{\sum }_{i=1}^{n}{A}_{i}}$$

Here, *n* is the number of faces, *ϕ*_*i*_ is the area-averaged variable value on face *i*, and *A*_*i*_ is the area of face *i*.

The displacement force acting on the stent graft can be divided into two parts: the force acting on the stent graft wall normally induced by blood pressure and the force acting on the graft wall tangentially that is induced by the WSS. By summing these force components, the displacement forces could be calculated. Numerical simulations and *in vitro* experiments have been widely adopted as two efficient methods of evaluating the displacement force, which can cause stent graft migration. Considerable research involving computational simulations has revealed that the displacement force is influenced by multiple factors, including the hemodynamics within the AAA and structural characteristics of the stent graft^18,28^. The stent graft displacement forces measured in *in vitro* experiments can be used as reference values for computational simulations. Therefore, an *in vitro* experiment was performed to investigate the effects of the “crossed limbs” strategy on the displacement force, as described in the “*In Vitro* Experiments” section.

## Results

**Figure 3:**
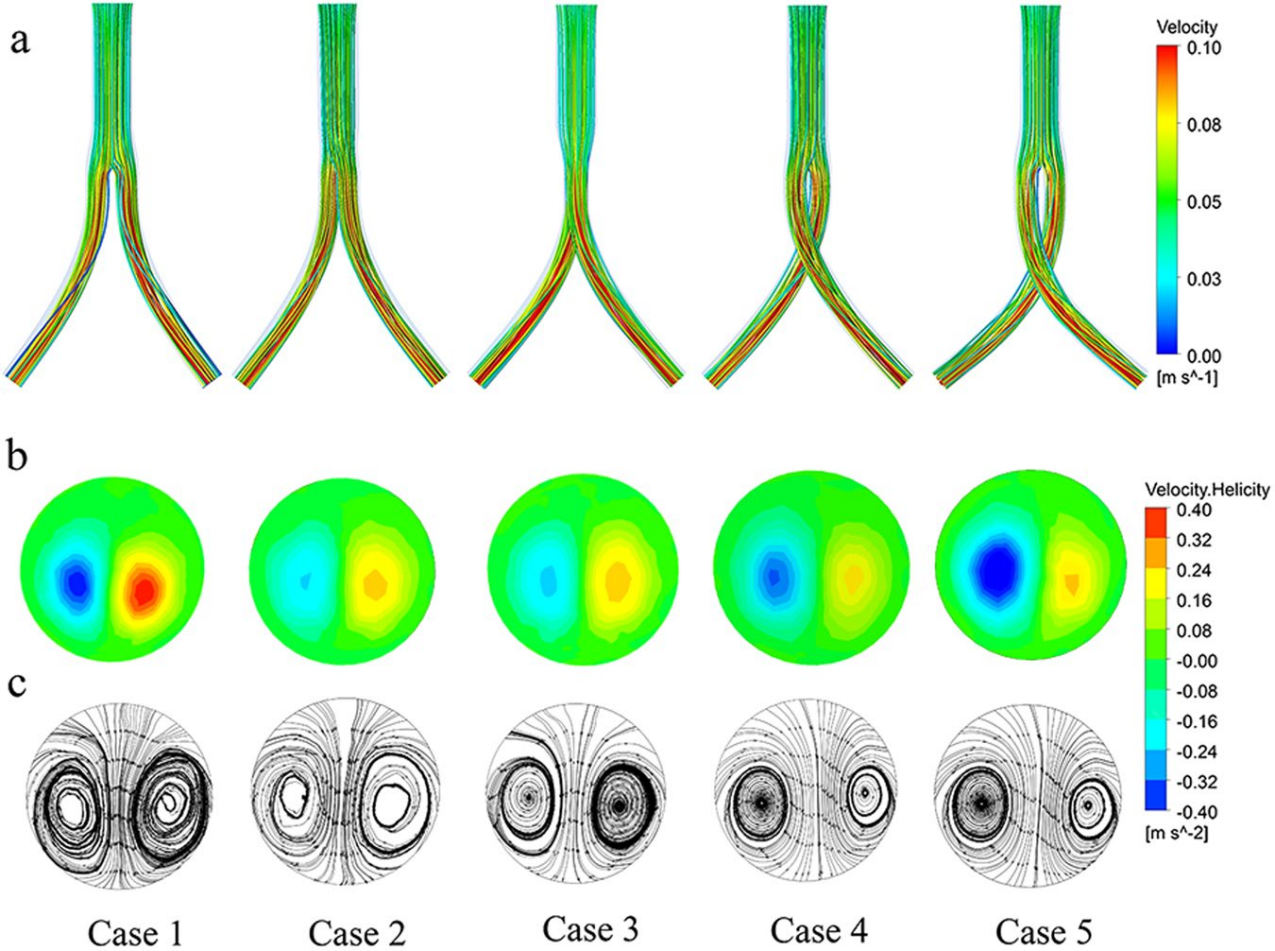
Steady-state flow patterns for all five cases. (**a**) Streamlines colored to indicate velocity magnitude; (**b**) helicity contours of the left outlet; (**c**) surface streamlines within the left outlet.

Figure 3 shows the steady-state velocity streamlines in all five cases; these streamlines are colored based on the velocity magnitude. The velocity streamline is higher within the iliac graft than in the trunk graft and twists along the iliac artery graft passageway. Two asymmetric helices are evident within the surface of the left iliac graft outlet. The left- and right-handed helical flow are equal in Cases 1–3, while in Cases 4 and 5, the left-handed helical flow is slightly greater than the right-handed flow.

**Figure 4:**
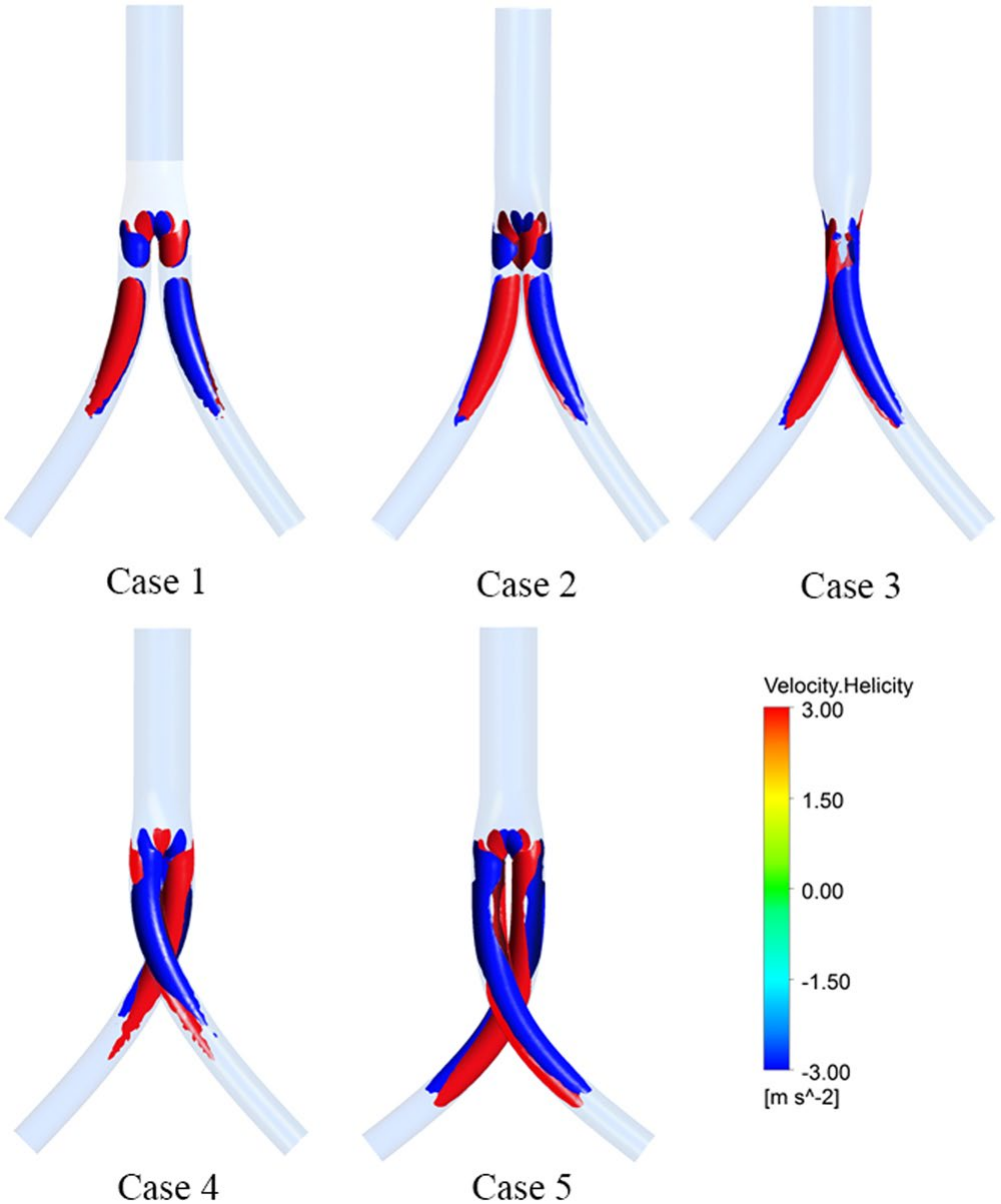
Helicity iso-surfaces for all five cases at the peak systole (*t* = 0.1 s). The helical structures with right-handed and left-handed rotation appear in red and blue, respectively.

The helical structure developed within the stent graft can be observed by displaying the helicity iso-surface. The helicity iso-surfaces corresponding to −3 m/s^2^ and 3 m/s^2^ at the peak systole (*t* = 0.1 s) are depicted in Fig. 4. In each case, the most remarkable hemodynamic feature is the distinct counter-rotating helical flow structure along the iliac graft. Moreover, as is clear from Fig. 4, with increasing torsion angle, the helical structures within the iliac artery grafts become more obvious.

**Figure 5:**
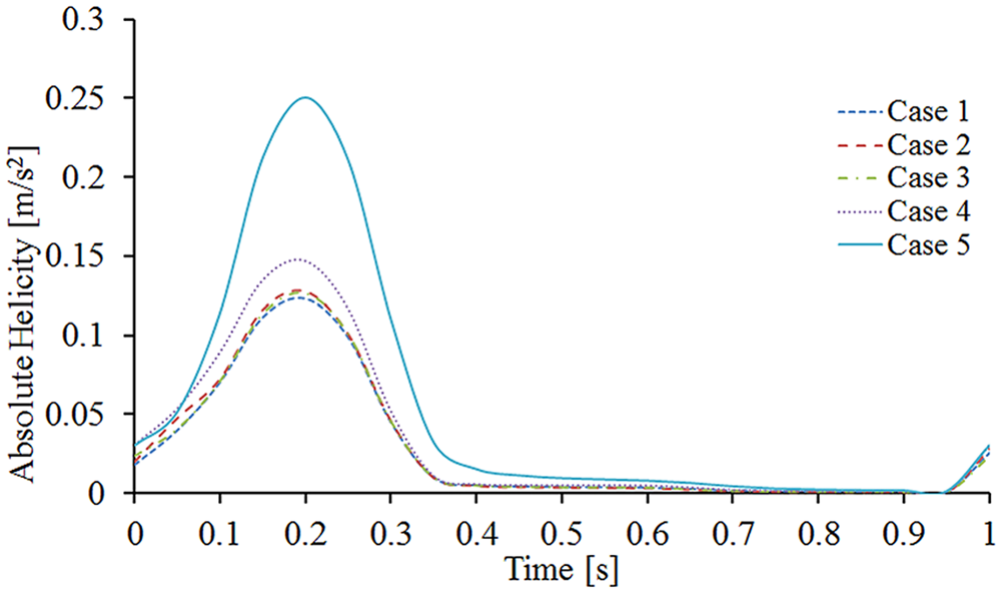
Variations of absolute helicity at the left outlet for all five cases.

Furthermore, as depicted in Fig. 5, the magnitude of the absolute helicity during the systole period is clearly higher than it is in the rest of the cardiac cycle. The absolute helicity variations in the left outlet exhibit almost no differences between the BSGs with torsion angles of 0° (Case 1), 45° (Case 2), and 90° (Case 3). When the torsion angle is 135° (Case 4), the variation of the absolute helicity increases, and when the torsion angle is 180° (Case 5), the left iliac outlet has the highest absolute helicity.

**Figure 6:**
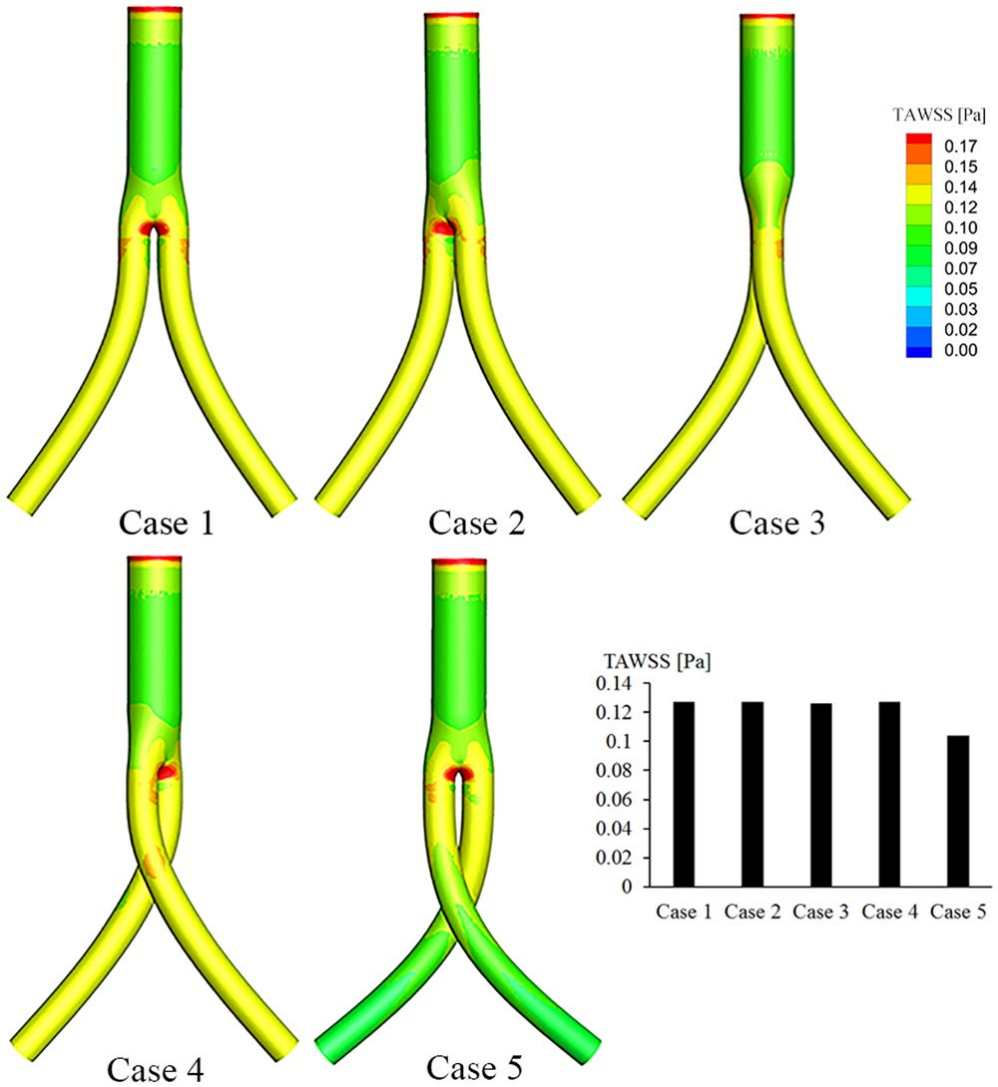
TAWSS contours based on pulsatile flow computations. The histogram shows the area-averaged mean TAWSSs along the iliac artery grafts for all five cases.

The TAWSS contours for both cases are depicted in Fig. 6. A relatively high TAWSS area can be observed in the bifurcated region of the BSG. In addition, the TAWSS in the iliac grafts is substantially decreased in Case 5 compared with the other cases. For analysis, the area-averaged TAWSSs on the iliac grafts for both cases were extracted and are presented as a histogram. The area-averaged TAWSS for the non-crossed stent graft (Case 1) is about 0.13 Pa. With increasing torsion angle, the area-averaged TAWSS decreases to almost half for Case 2 and stays nearly the same (0.13 Pa) in Cases 2–4; finally, for the totally crossed BSG (Case 5), it drops to 0.11 Pa.

**Figure 7:**
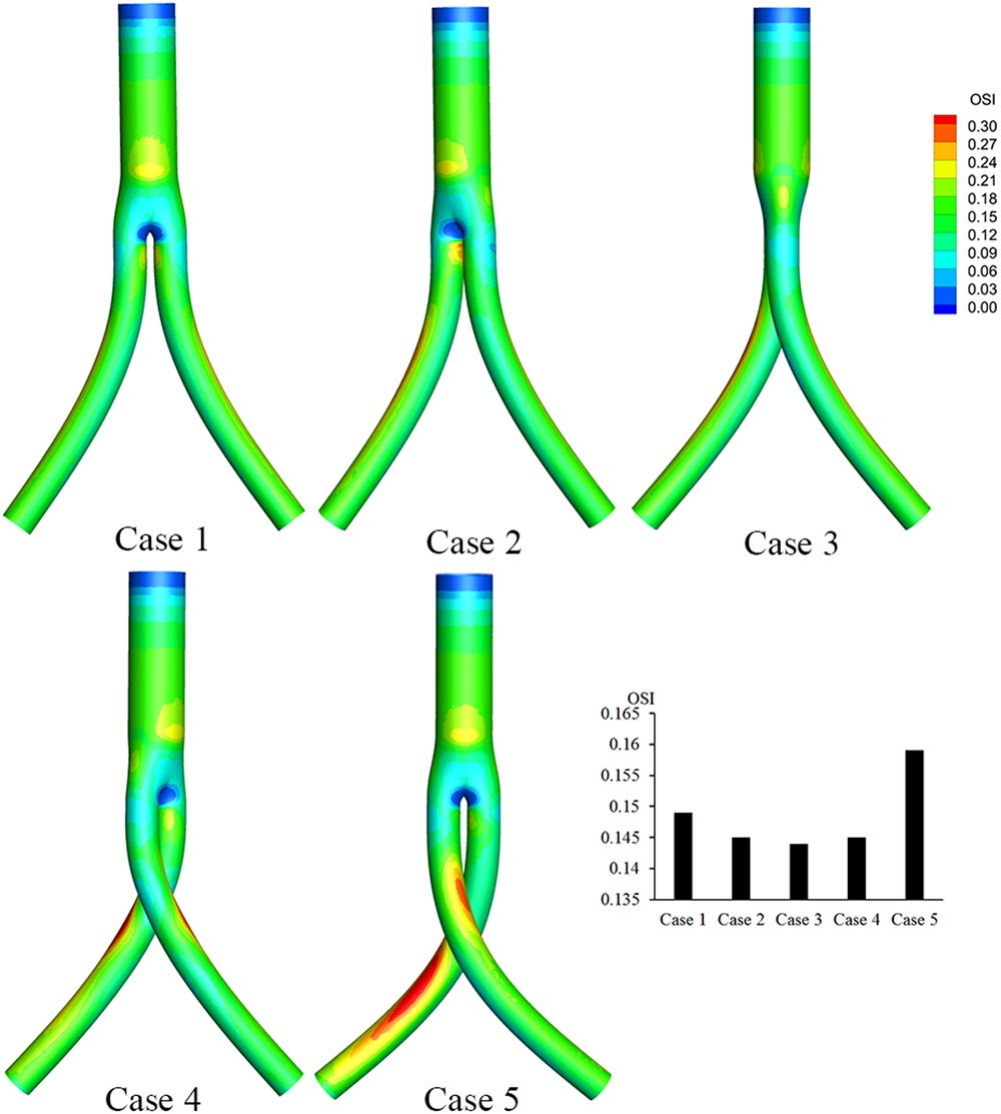
OSI contours based on pulsatile flow computations. The histogram shows the area-averaged mean OSIs along the iliac artery grafts for all five cases.

As depicted in Fig. 7, the bifurcated regions of the BSGs have relatively low OSI areas. The strip areas of high OSI along the iliac grafts are relatively large in Case 5 compared with the other cases. The area-averaged OSIs in the iliac grafts in both cases were extracted and compared using a bar graph. Our results indicate that the OSI first decreases with increasing torsion angle, then starts to increase when the torsion angle reaches 135°.

**Figure 8:**
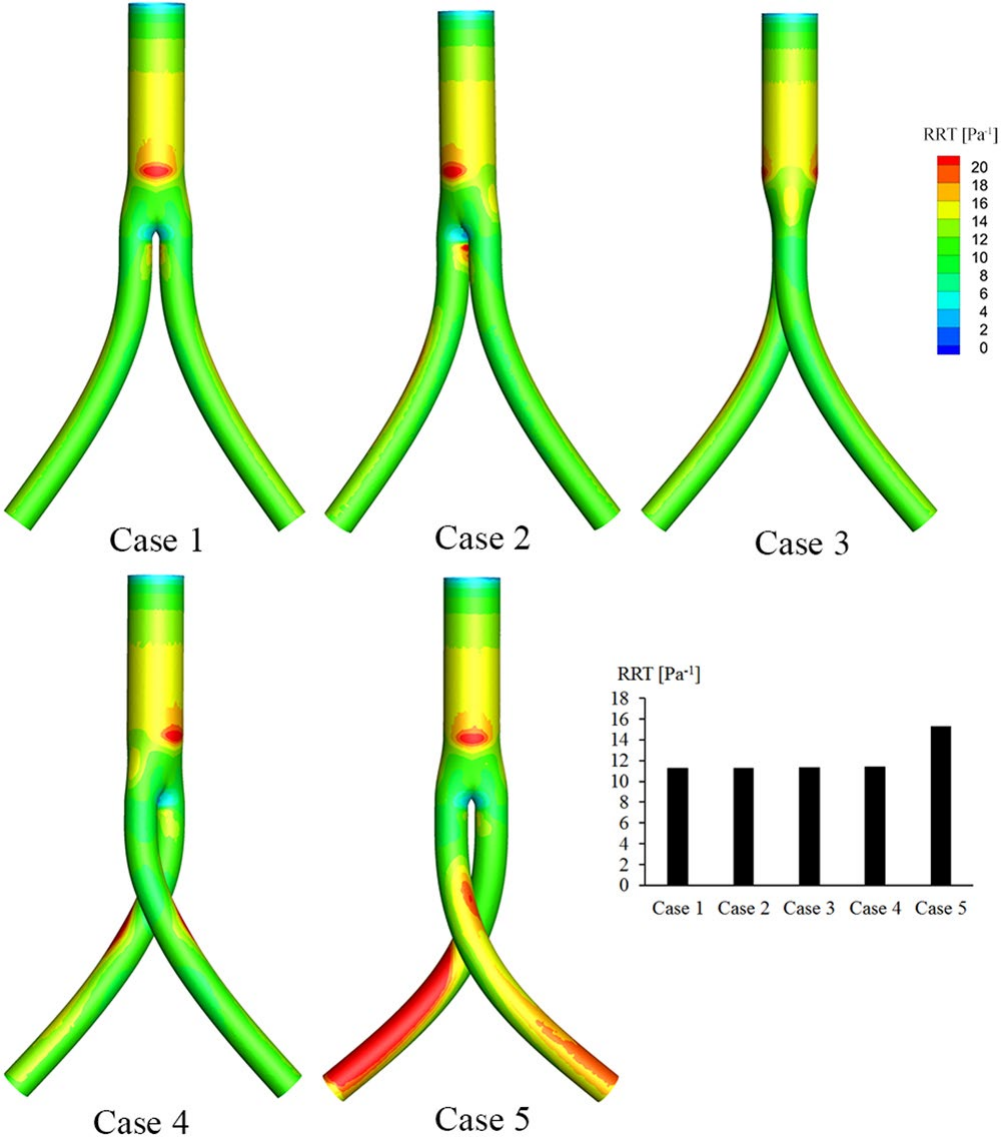
RRT contours based on pulsatile flow computations. The histogram shows the area-averaged mean RRTs along the iliac artery grafts for all the five cases.

Moreover, as shown in Fig. 8, the RRT distribution along the trunk of the BSG is similar in both cases. While the ribbon areas of relatively high RRT along the outer surfaces of iliac grafts are evident in Case 4, they are even more obvious in Case 5. The area-averaged weighted RRTs in the iliac grafts in both cases were extracted and compared using the histogram shown in Fig. 8. The area-averaged mean RRT increases from 6 Pa^−1^ to 15 Pa^−1^ as the torsion angle increases from 0° to 180°. In contrast, in Cases 2–4, the area-averaged mean RRT stays nearly the same and is higher than in Case 1, but lower than in Case 5. Examining the OSI and RRT together, it is clear that high RRT regions always correspond to high OSI regions.

**Figure 9:**
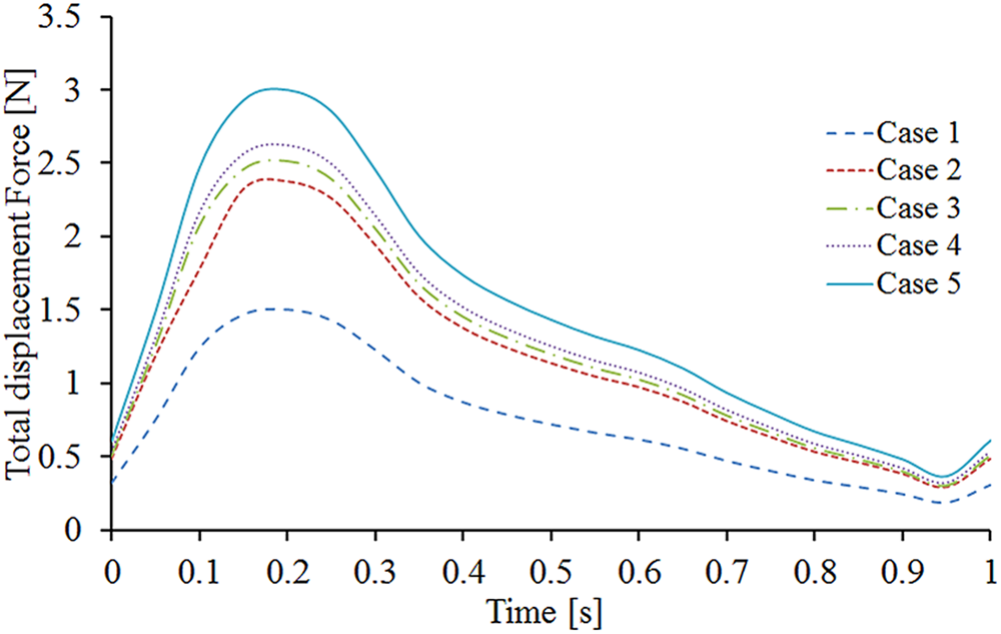
Displacement force variations for all the five cases.

As is evident from Fig. 9, the displacement force variations agree with the pressure waveform trends. The displacement force reaches its maximum when the pressure at the outlet reaches its peak value in the cardiac period. In case 1, the maximum displacement force reaches about 1.5 N when the torsion angle is 0° (Case 1), and it increases to 3 N in Case 5 when the torsion angle increases to 180°.

### *In Vitro* Experiments

**Figure 10:**
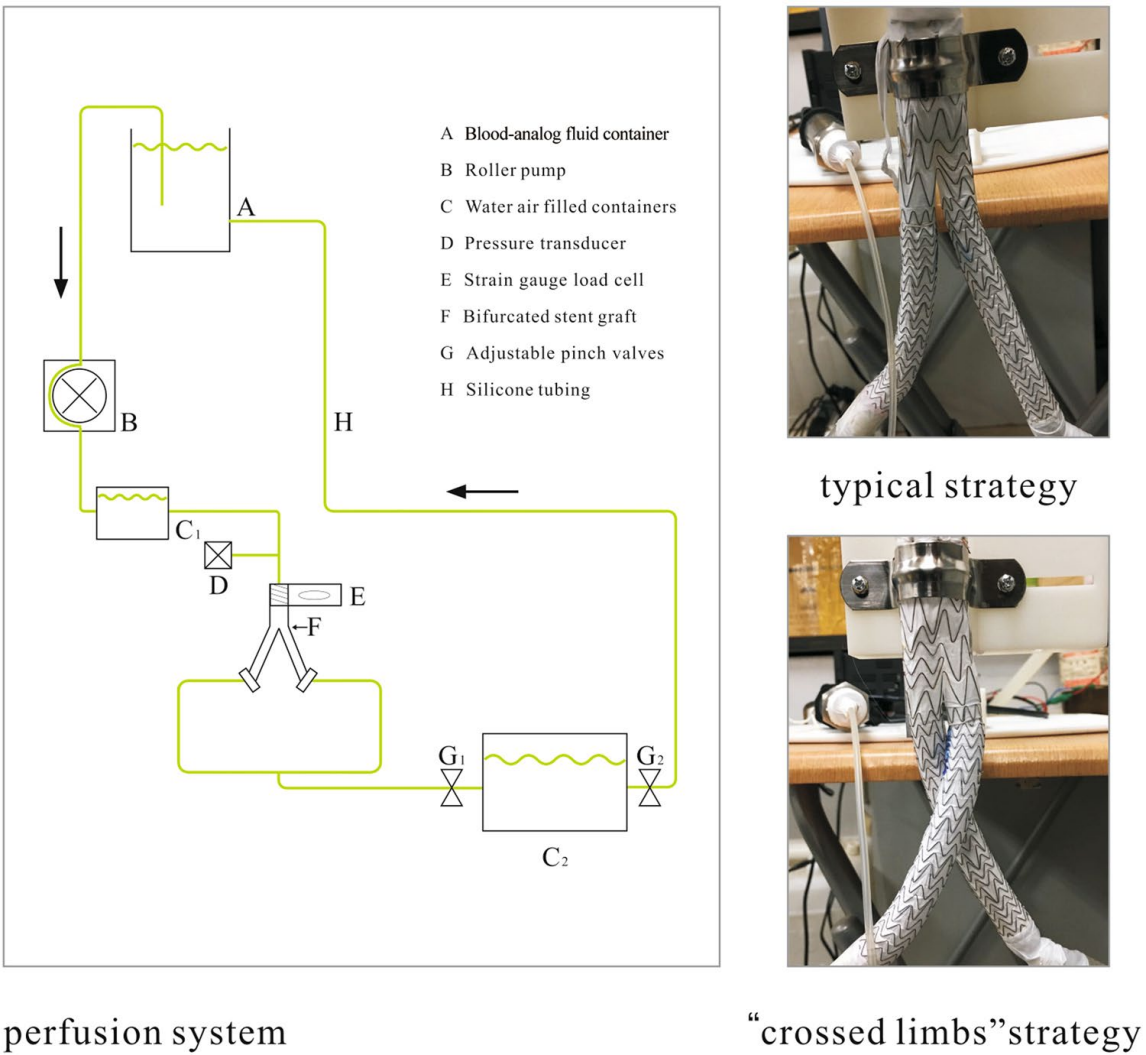
Schematic of the perfusion model and two BSG deployment strategies.

To evaluate the migration risk when the “crossed limbs” strategy is employed, we designed an experiment to measure the displacement force. As shown in Fig. 10, a perfusion system was built to provide a steady flow perfusion pressure within the BSG. The blood analog fluid used to mimic blood was prepared by mixing 33.3% by volume of glycerol with water at room temperature. The density and dynamic viscosity of this blood analog fluid remained about 1.05 g/cm^3^ and 0.0033 Pa · s, respectively, which are approximately equal to the corresponding values for blood. To imitate blood perfusion of the AAA, the blood analog fluid was then perfused in the fluid loop by a roller pump. Silicone tubes were connected to transport the blood analog fluid. Peripheral resistance was achieved by using the pinch valves connecting the water- and air-filled containers. To obtain a precise pressure within the stent graft, the water levels in the containers and pinch value adjustments needed to be controlled precisely.

A commercial BSG was used to perform the *in vitro* experiments. The diameter of the proximal entrance was 26 mm, and the diameter of the iliac part was 15 mm. The total length was 180 mm when the length of the iliac graft was 140 mm. The proximal part of the BSG was fastened to the strain gauge load cell with connectors. To ensure that the displacement force would be transferred to the load cell at the extreme, the stent graft was secured and deployed at the outer face of the load cell. Both ends of the stent graft were connected to the silicon tube using soft rubber tubes with highly elasticity that would not influence the displacement force measurements. The load cell measurements ranged from 0 N to 10 N; in addition, calibration was conducted before measurement. To monitor the perfusion pressure of the stent graft, a pressure transducer was deployed in the fluid loop; in addition, a force monitor was used to display and record the force. In particular, three perfusion pressure levels of 60, 80, and 100 mmHg were adopted to measure the displacement forces corresponding to torsion angles of 0°, 45°, 90°, 135°, and 180°. Before each test, it was necessary to confirm zero leveling of the BSG and to perform *in situ* calibration of the perfusion pressure. All of the measurements were performed when the flow was steady.

As shown in Table 2, the mean displacement force was evaluated in each case. The results of our *in vitro* experiments indicate that the displacement force increases with increasing pressure in both cases. The mean displacement force gradually increases as the torsion angle increased from 0° to 180° for each perfusion pressure level. The displacement force of the stent graft with 180° torsion is between 0.17 N and 0.26 N, higher than that in the case of 0° torsion. The displacement forces obtained in the simulations and *in vitro* experiments indicate that the trend of the displacement force is the same with increasing perfusion pressure and increasing torsion angle. Specifically, the displacement force increases with increasing torsion angle as well as with increasing perfusion pressure.

**Table 2:** Displacement forces (N) corresponding to various torsion angles and perfusion pressures.

Torsion angle	0°	45°	90°	135°	180°
60 mmHg	1.47	1.52	1.55	1.59	1.64
80 mmHg	1.63	1.65	1.69	1.72	1.77
100 mmHg	1.92	1.96	2.02	2.11	2.18

## Discussion

The deployment of “crossed limbs” for AAA treatment has been widely accepted in cases of unfavorable aneurysm neck angulation or AAAs with widely splayed common iliac arteries^5,6^. In our study, we constructed a series of models for the “crossed limbs” surgery strategy with different torsion angles and evaluated their hemodynamic characteristics by analyzing the results of numerical simulations and *in vitro* experiments.

Our results reveal that the “crossed limbs” approach induces double helical blood flows at both outlets of the limb graft. In the study by Shek *et al*.^3,6^, only one of the outlets was found to have a double helical flow, while the flow at the other outlet had the structure of single helical flow. The main differences between our results and theirs are due to the use of different models. The models we used were symmetrical, enabling the determination of common features of this specific strategy, whereas theirs were idealized stent graft models based on patient-specific medical data, which could provide personalized features. Nevertheless, based on the results of our study and those obtained by Shek *et al*.^3,6^, it can be concluded that the “crossed limbs” strategy can induce helical flows in a graft whether or not the model is symmetrical. In addition, our findings demonstrate that the larger the torsion angles of the limbs, the stronger the helical flow. Therefore, based on the results of our study and the study by Shek *et al*.^3,6^ we believe that the “crossed limbs” strategy can be beneficial for AAA treatment in terms of helical flow because, as mentioned earlier, helical flow may protect the arteries by suppressing the accumulation of atherogenic low-density lipoproteins within the arterial wall^8^, thus enhancing the O_2_ supply to the arterial wall^29^ and reducing platelet/monocytes adhesion^14,15^. Furthermore, because the intensity of the helical flow produced by the “crossed limbs” strategy increases with increasing torsion angle and a 180° torsion angle can produce the strongest helical flow, we suggest that the largest possible torsion angle could be employed during surgery.

However, although the “crossed limbs” strategy produces helical flow at both stent graft outlets, which could be advantageous, our results also show that other flow parameters could worsen. For instance, the TAWSS in the stent graft was lower that in the direct stent graft configuration (Case 1), and the RRT increased with increasing torsion angle. In particular, for the 180° torsion angle case (Case 5), the changes in the TAWSS and RRT were sharp compared with those in Case 1. Meanwhile, the OSI first decreased with increasing torsion angle, then started to increase when the torsion angle reached 135°. This phenomenon can be ascribed to the appearance of ribbon areas with relatively low TAWSSs and high RRTs along the distal parts of the stent graft in Case 5. It has been widely accepted that a low WSS is usually associated with blood flow stagnation and thrombus formation^30,31^, and a high OSI and RRT, which are derived from the WSS, lead to thrombosis by stimulating platelet aggregation, thus activating platelets and increasing the residence time of procoagulant microparticles^32–34^. Therefore, the ribbon areas appearing along the distal parts of stent grafts may be prone to thrombus formation and need to be paid more attention when employing the “crossed limbs” strategy.

In clinical practice, stent graft migration is a general post-operative complication caused by the hemodynamic loads acting on the stent graft. Our results indicate that the displacement force variation follows the trend of the pressure waveform, which is consistent with the results of previous studies^18,19^. Li *et al*. demonstrated that several factors could affect the migration behavior, such as the iliac bifurcation angle and perfusion pressure within the stent graft^18,28^. The results of both numerical and *in vitro* experiments revealed that the displacement force increases as the perfusion pressure increases, indicating that the pressure influences the risk of stent graft migration. Furthermore, the displacement force increases with increasing bilateral iliac graft torsion angle. Therefore, our results indicate that the stent graft torsion angle also regulates graft migration. Rahmani *et al*. conducted *in vitro* experiments to evaluate the pullout forces of various stent grafts, which are the threshold forces required for displacement^20^. Their results revealed that the pullout force decreases with increasing off-axis angulation. By considering the decrease of the pullout force and the increase of the migration force acting on the stent graft due to the increased torsion angle, the risk of BSG migration certainly increases if the “crossed limbs” strategy is adopted to treat AAAs. Previous researchers have reported that the dislodgement forces that endografts can withstand before migration range from 6.5 N to 26.5 N^35^. Meanwhile, the maximum displacement force measured in the simulations in the present study was about 3 N, which is much lower than the actual force required for stent graft migration. Therefore, changing the torsion angle in the “crossed limbs” strategy may not induce BSG migration in the short term. However, as migration is a gradual process, the risk of migration still needs to be paid much more attention in the long term when the “crossed limbs” strategy is employed.

It is important to note that our study was limited in that, for simplicity, we used idealized geometric models for the “crossed limbs” strategy of AAA repair. Previously reported BSG parameters were used as references for the idealized stent graft generated in our study; thus, the common hemodynamic features present when different deployment strategies are utilized could be obtained^18,19^. Another limitation is that the stent graft wall was regarded as rigid. However, since graft deformation under blood pressure was not apparent, this assumption is still valid. The numerical results obtained in previous studies^17,19^ support the validity of this assumption. Morbiducci *et al*. concluded that the hemodynamics in the aorta, including disturbed shear and bulk flow structures, may be misleading when an idealized velocity profile is imposed as the inlet conditions^10^. Youssefi *et al*. also demonstrated that the hemodynamic evolution in the aorta becomes inaccurate when idealized inflow velocity profiles are used^36^. Therefore, the imposition of idealized flat inlet conditions could be another limitation of the present study. However, as the blood flow within the human arteries could not be fully developed and a flat velocity was imposed in all of the models for comparison among the configurations with different torsion angles, the conclusions derived from our results would not be influenced. It should also be mentioned that it was difficult to replicate the pulsatile pressure conditions in the *in vitro* experiments. Therefore, we set three pressure levels in the pulsatile pressure waveform to clarify the influences of the torsion angle and perfusion pressure on the displacement forces. In a pilot study, these simplifications may affect the accuracy of the numerical results; however, they may not influence the primary conclusions regarding the hemodynamic performance of the “crossed limbs” strategy.

## Conclusions

This study revealed that the “crossed limbs” strategy can be beneficial for AAA repair with respect to helical flow. However, unfavorable hemodynamic performance was observed by evaluating the typical hemodynamic parameters, including the TAWSS, OSI, and RRT. In addition, the risk of stent graft migration is increased, which was verified by performing numerical simulations and *in vitro* experiments. Therefore, one should use caution when performing the “crossed limbs” strategy during surgery. Our results are preliminary findings, and more research is necessary to achieve a better understanding of the “crossed limbs” strategy in clinical practice.
